# Virtual Reality Is Safe and Can Reduce In‐Hospital Anxiety and Pain: A Systematic Review With Meta‐Analyses and Trial Sequence Analyses

**DOI:** 10.1002/ejp.70165

**Published:** 2025-11-05

**Authors:** Karsten L. Lassen, Kristian Hermander, Pether Jildenstål, Nanna Wagner, Annelie Augustinsson, Carina Sjöberg, Anja Geisler

**Affiliations:** ^1^ Department of Health Sciences Lund University Lund Sweden; ^2^ Department of Urology Zealand University Hospital Roskilde Denmark; ^3^ Department of Anaesthesiology, Surgery and Intensive Care Medicine Sahlgrenska University Hospital Gothenburg Sweden; ^4^ Institute of Health and Care Sciences, Sahlgrenska Academy, University of Gothenburg Gothenburg Sweden; ^5^ Faculty of Nursing and Health Sciences Nord University Namsos Norway; ^6^ Department of Anesthesiology and Intensive Care Skane University Hospital Lund Sweden; ^7^ Department of Anesthesiology and Intensive Care, Hospital and School of Medical Sciences Örebro University Örebro Sweden; ^8^ Department of Digestive Diseases, Transplantation and General Surgery Rigshospitalet Copenhagen Denmark; ^9^ Department of Clinical Medicine, Faculty of Health and Medical Sciences Copenhagen University Copenhagen Denmark

## Abstract

**Background and Objective:**

Virtual reality (VR) is a rapidly evolving technology that is currently utilized in hospital settings for various types of surgical procedures. The extent to which VR is evident in improving patient outcomes is unknown. This systematic review assesses the impact of VR on adult patients undergoing elective surgical procedures.

**Databases and Data Treatment:**

The following databases were sought: CENTRAL, MEDLINE, EMBASE, and CINAHL. All studies published after 2017 were included. The risk of bias was assessed using the ROB2 and ROBINS‐I. Meta‐analyses and Trial Sequential Analyses were performed, and the quality of evidence was evaluated using the GRADE approach for the randomised controlled trials.

**Results:**

A total of 37 full‐text studies (*n* = 3152) were included. VR significantly reduced anxiety measured by the Numeric Rating Scale (*p* < 0.0001) and the State–Trait Anxiety Inventory (*p* = 0.008). Furthermore, Numeric Rating Scale pain was significantly reduced (*p* < 0.00005), with a significantly shorter recovery time and a non‐significant improvement in patient satisfaction. Adverse events were infrequent and mild, with no serious adverse events reported. The risk of bias was primarily “some concerns”, and the certainty of evidence ranged from moderate to low.

**Conclusions:**

VR appears effective in reducing pain and anxiety in adult patients in an in‐hospital setting. It offers a relatively safe adjunct to standard care with minimal side effects. However, heterogeneity in outcomes and the risk of bias suggest a need for more standardised, high‐quality trials.

**Significance Statement:**

This systematic review with meta‐analysis and trial sequential analysis provides updated evidence that virtual reality can significantly reduce anxiety and pain in patients undergoing surgical procedures. Through combining recent RCTs and cohort studies with robust methodological approaches, this review strengthens the evidence for VR as an effective non‐pharmacological intervention. With minimal adverse events and significant improvements in recovery time, VR represents a scalable tool that can strengthen multimodal strategies and promote safer and more comfortable patient experiences.

## 1 Introduction

Virtual reality (VR) was first introduced in healthcare during the late 1990s, primarily within the domains of surgical training and rehabilitation, where it offered a novel method for simulation and recovery (Satava 1993). VR technology enables users to view, interact with, and immerse themselves in a computer‐generated virtual environment. This can be achieved with an occlusive head‐mounted display that projects a virtual view and provides noise‐cancelling audio. The underlying mechanism for the distraction effect of VR in reducing unpleasant experiences may be explained by the limited capacity of the human brain to process large amounts of sensory input, which can thereby divert attention away from unpleasant stimuli (Ahmadpour et al. 2019; Chow et al. 2021; Hoffman et al. 2007). Technological advancements, have expanded the scope of VR, enabling its integration across various clinical contexts, including wound care and burn dressing procedures, but also in psychiatric care (Bhugaonkar et al. 2022; Chirico et al. 2016; Garrett et al. 2014; Wiederhold et al. 2014).

Up to 80% of patients experience anxiety in relation to their surgical procedures (Abate et al. 2020; Bedaso et al. 2022; Dong et al. 2022; Flores et al. 2023). Anxiety can be triggered by concerns about anaesthesia, potential complications, pain, or uncertainty regarding the outcome of the procedure. Anxiety has postoperative consequences such as affected vital signs, nausea, increased pain, and insomnia (Abate et al. 2020). Instead of providing patients with sedatives, which are associated with an increased risk of cognitive and postoperative side effects, particularly in elderly or vulnerable patients non‐pharmacological approaches have been suggested. These include music therapy, guided relaxation, and cognitive‐behavioural strategies, which have shown effects in clinical trials to decrease anxiety, pain and dissatisfaction (Cakmak et al. 2017; Li et al. 2011). However, their effectiveness is often limited by individual preferences, setting constraints, and the need for active participation. Here, VR could be useful as an alternative or supplement to the pharmaceuticals, not only regarding decreasing anxiety but also levels of pain (Kodvavi et al. 2023). Postoperative pain is a challenge in modern healthcare today since nearly 20% of patients endure severe pain (Small and Laycock 2020). Despite advances in surgical techniques and pain management strategies, a large proportion of patients continue to experience inadequately controlled pain following surgery. This issue affects not only patient comfort but also has far‐reaching consequences for the recovery process. Numerous benefits have been suggested in relation to the use of VR, including stress reduction, increased patient satisfaction, and improved patient engagement, as frequently emphasised in the existing literature (Chang et al. 2021; Chiu et al. 2023; Shepherd et al. 2022). However, a comprehensive comparison of all the research in this area has not yet been conducted to provide actual evidence for the use of VR in elective surgery for patient outcomes such as anxiety, pain, patient readiness, and patient satisfaction. Although VR technology is generally regarded as safe, users may also experience harm, such as nausea or fatigue, commonly referred to as cybersickness (Simón‐Vicente et al. 2024), this field also needs to be taken into consideration.

Therefore, a systematic review of existing literature is warranted to evaluate the benefits and potential harms of VR in hospital settings, with a focus on its effects on anxiety and pain.

## 2 Methods

This systematic review adheres to the methodological framework validated by the Cochrane Collaboration, version 22 August 2019 (Higgins et al. 2011), and has been conducted in alignment with the Preferred Reporting Items for Systematic Reviews and Meta‐Analyses (PRISMA) guidelines (Rethlefsen et al. 2021). Before the literature search was conducted, the protocol was registered in the International Prospective Register of Systematic Reviews (PROSPERO) on 15 April 2023, under registration number CRD42023414321.

### 2.1 Search Strategy

In collaboration with a professional search coordinator, a comprehensive search strategy was developed, encompassing Medical Subject Headings (MeSH) and terms from all fields to ensure the inclusion of the relevant trials (Appendix S1). All full‐text studies published at The Cochrane Library's CENTRAL, MEDLINE, EMBASE, and CINAHL were sought. The last search was conducted on 19 November 2024. Additionally, reference lists from systematic reviews and relevant articles were manually screened for eligible trials, and non‐indexed publications from Google Scholar (limited to the first 500 hits) were also examined (see Appendix S1).

### 2.2 Outcomes

The primary outcome was the level of anxiety during the elective surgical procedure. Secondary outcomes included: pain levels, readiness (defined as the patient's cognitive, emotional, and behavioral capacity to engage with and participate in the surgical process, ultimately influencing surgical outcomes), patient satisfaction, adverse events, serious adverse events, time spent in the Post Anaesthesia Care Unit (PACU), and surgery delay.

### 2.3 In‐ and Exclusion Criteria

The inclusion criteria were patients ≥ 18 years, studies investigating the primary or secondary outcome in a hospital setting, studies using VR as a 3‐dimensional computer‐animated environment displayed in surround stereoscopic vision placed on the head (HMD), and studies using 360‐degree videos displayed on a VR HMD. Due to the fast development of VR, only studies published after 2017 were considered for inclusion. The intervention was required to be initiated in the immediate operative period, and the studies had to report at least one of the predefined outcomes. Exclusion criteria were systematic reviews, abstracts, protocols, unpublished observations, quasi‐randomised studies, articles not written in English, participants under 18 years, studies conducted in a psychiatric setting, editorials, letters, protocol articles, and comments.

### 2.4 Selection Process and Data Collection

Six authors independently screened titles and abstracts by using the Covidence systematic review software (Veritas Health Innovation, Melbourne, Australia) to determine suitability based on the predefined inclusion and exclusion criteria, and thereafter, the full‐text screening was performed. Two authors (KLL and KH) performed the evaluations individually and then compared their results in pairs. If conflicts arose, the senior researcher, AG, resolved the issue. Reasons for study exclusions were documented at the full‐text level. When data were missing or bias assessments were unclear, the corresponding authors were contacted via email. A follow‐up was sent after 2 weeks if no response was received. Open‐ended questions were used to reduce the risk of confirmation bias.

### 2.5 Risk of Bias Assessment

Following the guidance by Cochrane (Rethlefsen et al. 2021), the Risk of Bias assessment was performed individually and compared in pairs. Randomised trials were evaluated utilising the “Revised Cochrane Risk‐Of‐Bias Tool for Randomized Trials” (ROB2) (Higgins et al. 2011). Non‐randomised cohort studies were evaluated following “Risk of Bias in Non‐randomized Studies of Interventions” (ROBINS‐I) (Sterne et al. 2016). In accordance with the ROB2 guidance, the overall risk of bias for each study was determined by the highest risk rating in any individual domain: a study was judged to be “low risk of bias” if all domains were rated as “low”; “some concerns”, if at least one domain raised some concerns, and “high risk” if at least one domain was rated high or multiple domains, raised some concerns. A similar principle was applied for ROBINS‐I, where the overall judgement reflects the most severe level of bias observed across the assessed domains.

### 2.6 Statistics

Pain and anxiety scores, rated by Visual Analog Scale (VAS) (0–100/0–10) or Numerical Rating Scale (NRS) (0–100/0–10), were all converted into a 0–10 scale. The mean values and standard deviations (SDs) from intervention groups were aggregated for trials featuring multiple treatment arms, and the control group was divided equally by the number of intervention arms. If data were presented in median values and interquartile range, they were converted to mean and SDs following the methodology proposed by Hozo et al. (2005). Statistical analyses involved conducting meta‐analyses and sensitivity analyses using Review Manager (RevMan version 5.4.1) whenever two or more trials reported the preplanned outcomes concerning continuous data, such as anxiety, pain, readiness, patient satisfaction, time in PACU, and nausea, using the same approach. Trial sequential analysis (TSA) (Wetterslev et al. 2017) was executed using version 0.9.5.10 Beta software (Copenhagen Trial Unit, Center for Clinical Intervention Research, Rigshospitalet, Copenhagen, Denmark). Heterogeneity among trials was evaluated using the *I* ^2^, which quantifies the variance observed. Forest plots were visually inspected to assess statistical heterogeneity. Sensitivity analyses were conducted to investigate whether the choice of summary statistics and decisions made during the review process influenced the meta‐analysis conclusions. TSA was applied to the primary and secondary outcomes to account for random errors. The diversity‐adjusted required information size (DARIS), alongside the cumulative Z‐curve, was then calculated and visualised. TSA could not be executed if the accumulated information size was less than 5% or if the data were insufficient. Funnel plots were created when 10 or more trials were included in the meta‐analysis to evaluate heterogeneity using the *I* ^2^ statistic and forest plots. Hence, a mean difference of 1 (NRS (0–10)) for pain and anxiety scores was established as the threshold. The Grading of Recommendations, Assessment, Development, and Evaluation (GRADEpro GDT) framework was used to assess the certainty of evidence in the RCTs. Outcomes from non‐randomised studies were reported separately in narratives.

## 3 Results

**FIGURE 1:**
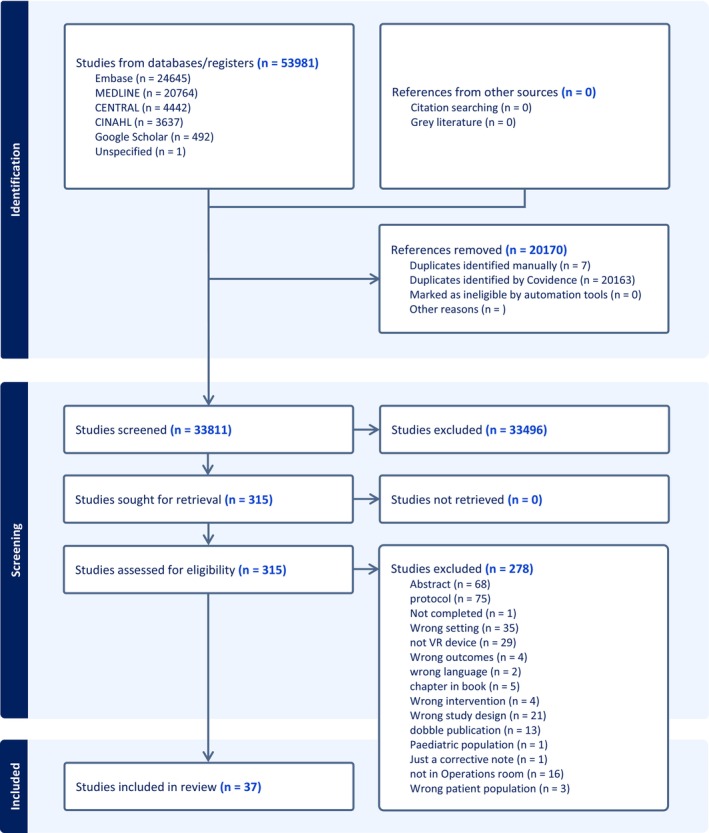
PRISMA (Preferred Reporting Items for Systematic Reviews and Meta‐Analyses) flow diagram illustrating the study selection process.

From the literature search, 33,811 studies were identified and screened based on their titles and abstracts. Of these, 33,496 were excluded according to the inclusion and exclusion criteria. Following a full‐text assessment, 278 articles were excluded primarily because VR was provided with smartphones and incorrect hospital settings, resulting in 37 studies being selected for the final extraction. (Figure 1). After the data extraction process, it was possible to perform meta‐analyses for six groups: anxiety, pain, satisfaction, PACU time, adverse events, and serious adverse events. Trials included are presented in Table 1. Trials in meta‐analyses are summarized in Table 2. No studies reported on outcomes regarding readiness or surgical delay. Sensitivity analyses were performed but showed no differences.

**TABLE 1:** Summary of the key characteristics of the included studies.

Authors	Region	Study design	Aetiology	Anxiety	Satisfaction	Pain	Time	AE^j^/SAE^k^
Alaterre et al. (2020)	France	Cohort	Orthopaedic surgery, upper limb surgery	NRS^c^	NRS^c^	NA^i^	VR Group: 25 [20; 30] minutes Standard Care Group: 29 [20; 31] minutes	No nausea was reported SAE^k^, NA^i^
Almedhesh et al. (2022)	Saudi Arabia	RCT^a^	Obstetrics, caesarean section	NVFAS^e^	BSS‐R^f^	NA^i^	NA^i^	NA^i^
Arifin et al. (2023)	Indonesia	RCT^a^	Lower abdominal or lower extremity surgery	STAI^d^	Likert scale	NA^i^	NA^i^	No significant difference SAE^k^, NA^i^
Barry et al. (2022)	USA	Cohort	Orthopaedic surgery, total hip arthroplasty (THA) and total knee arthroplasty (TKA)	NA^i^	NA^i^	NRS^c^	IVR: 1.7 h, No IVR: 2.3 h	Similar nausea levels SAE^k^, NA^i^
Boonreunya et al. (2022)	Thailand	RCT^a^	Gastroenterology	NAi	Likert scale	NRS^c^	NA^i^	No significant difference SAE^k^, NA^i^
Bruno et al. (2020)	Germany	RCT^a^	Cardiology, transcatheter aortic valve implantation	VAS^b^	Questionnaire	NRS^c^	NA^i^	No significant difference SEA^k^, NA^i^
Carella et al. (2024)	Belgium	RCT^a^	Orthopaedic surgery, total knee arthroplasty	NA^i^	NA^i^	NRS^c^	Group VRH: 20.5 min Group Control: 62.5 min (median)	NA^i^
Faruki et al. (2022)	United States	RCT^a^	Orthopaedic surgery, hand surgery	NRS^c^	NRS^c^	NRS^c^	VR group: 53.0 min (IQR 43.0–72.0) Control group: 75.0 min (IQR 57.5–89.0)	NA^i^
Fouks et al. (2022)	Israel	RCT^a^	Gynaecology, specifically focused on hysteroscopy procedures	Questionnaire	NA^i^	NRS^c^	NA^i^	Control group: 6 (15.7%), VR group: 7 (15.9%) SAE^k^, NA^i^
Ganry et al. (2018)	France	Cohort	Oral and maxillofacial surgery	VAS^i^	NA^i^	NA^i^	NA^i^	NA^i^
Gökçe and Arslan (2023)	Turkey	RCT^a^	Cardiology, coronary angiography	STAI^d^	PCS^h^	NRS^c^	NA^i^	NA^i^
Gray et al. (2021)	United States	RCT^a^	Otolaryngology nasal endoscopy and debridement procedures	SUDS^g^	Likert scale	NA^i^	NA^i^	NA^i^
Hecken et al. (2023)	Germany	RCT^a^	Gynaecology. colposcopy	NRS^c^	NRS^c^	NRS^c^	NA^i^	NA^i^
Ko et al. (2024)	Hong Kong, China	RCT^a^	Emergency medicine, wound closure by suturing	STAI^d^	NRS^c^	NRS^c^	NA^i^	NA^i^
Lachkar et al. (2022)	France	Cohort	Bronchoscopy	VAS^b^	NRS^c^	NA^i^	NA^i^	NA^i^
Lind et al. (2023)	Germany	RCT^a^	Cardiology, transcatheter aortic valve replacement	STAI^d^	Recommended Yes/no	NRS^c^	NA^i^	No significant difference SAE^k^, NA^i^
Liu et al. (2022)	China	RCT^a^	Gastroenterology, colonoscopy	NA^i^	NA^i^	NRS^c^	NA^i^	NA^i^
Luczak et al. (2021)	Poland	RCT^a^	Urology, cystoscopy	NA^i^	NA^i^	NRS^c^	NA^i^	The use of VR sets was associated with higher levels of nausea SAE^k^, NA^i^
McCullough et al. (2023)	United States	Cohort	Orthopaedic, hand surgery	STAI^d^	Likert scale	NA^i^	NA^i^	NA^i^
Melcer et al. (2021)	Israel	RCT^a^	Obstetrics & Gynaecology amniocentesis	STAI^d^	NA^i^	NRS^c^	NA^i^	Two participants in the VR group experienced nausea
Palte et al. 2024	United States	RCT^a^	Gastroenterology, high‐resolution oesophageal manometry (HRM)	STAI^d^	NA^i^	McGill^n^	NA^i^	NA^i^
Pelazas‐Hernandez et al. (2023)	Spain	RCT^a^	Gynaecology, hysteroscopy	NAi	NA^i^	NRS^c^	NA^i^	Nausea is mentioned as a side effect commonly related to vagal reactions during hysteroscopy SAEk, NA
Perenic et al. (2023)	France	Cohort	Urology—transrectal MRI‐guided prostate biopsy	NA^i^	NA^i^	NRS^c^	NAi	No patients were concerned about nausea. SAE^k^, NA^i^
Peuchot et al. (2021)	France	Cohort	Orthopaedics Total knee arthroplasty	STAI^d^	EVAN LR^m^	NRS^c^	NA^i^	Nausea 10% in group 1 (VR) compared to 0% in group 2 (control) SAE^k^, NA^i^
Prabhu et al. (2024)	United States	RCT^a^	US‐guided core needle breast biopsies.	STAI^d^	Likert scale	NRS^c^	NA^i^	NA^i^
Rosielle et al. (2024)	Netherlands	RCT^a^	Hysterosalpingo‐graphy	APAIS^l^	Likert scale	NRS^c^	NA^i^	No significant differences
Sargut et al. (2024)	Germany	RCT^a^	Port implantation	NA^i^	NA^i^	McGill^n^	NA^i^	NA^i^
Schaake et al. (2024)	United States	RCT^a^	Thyroid biopsies and peripherally inserted central catheter placements	NA^i^	NAi	Adjusted NRS^c^	NA^i^	NA^i^
Sewell et al. (2023)	United Kingdom	RCT^a^	Gynaecology, hysteroscopy	NRS^c^	NA^i^	NRS^c^	NA^i^	VR group, two patients reported mild nausea SAE^k^, NA^i^
Shamali et al. (2024)	Denmark	RCT^a^	Gastroenterology, colonoscopy	STAI^d^	NRS^c^	NRS^c^	NA^i^	NA^i^
Sooriyaghandan et al. (2023)	Malaysia	RCT^a^	Bronchoscopy	STAI^d^	Likert scale	NRS^c^	NA^i^	NA^i^
Soret et al. (2022)	France	Cohort	Sternal bone marrow aspiration	Questionnaire	NA^i^	NRS^c^	NA^i^	NA^i^
Squara et al. (2024)	France	RCT^a^	Pacemaker or implantable cardioverter defibrillator implantation procedures	NRS^c^	NRS^c^	NRS^c^	NA^i^	13% in the VR‐Group encountered symptoms of cybersickness SAE^k^, NA^i^
Steinkraus et al. (2024)	Germany	RCT^a^	Port implantation procedures	STAI^d^	Likert scale	NRS^c^	NA^i^	No “VR” group, (3.9%). “VR” group, (0%) reported nausea SAE^k^, NA^i^
Veisman et al. (2024)	Israel	Cohort	Gastroenterology, colonoscopy	NA^i^	NRS^c^	NRS^c^	VR group: 65 min (median), Control group: 159 min (median)	No significant difference SAE^k^, NA^i^
Verain et al. (2024)	France	RCT^a^	Cardiology—coronary angiography and peripheral angioplasty	STAI^d^	Likert scale	Adjusted NRS^c^	NA^i^	There were no reports of AE or SAE^k^
Xu et al. (2024)	China	RCT^a^	Elective caesarean section	NRS^c^	NRS^c^	NA^i^	NA^i^	No significant difference SAE^k^, NA^i^

^a^ Randomised control trial.

^b^ Visual Analogue Scale.

^c^ Numeric Rating Scale.

^d^ State–Trait anxiety inventory.

^e^ Novel Visual Facial Anxiety Scale.

^f^ Behavioural Signs Scale – Revised.

^g^ Subjective Units of Distress Scale.

^h^ Perianesthesia Comfort Scale.

^i^ Not applicable.

^j^ Adverse event.

^k^ Serious adverse event.

^l^ Amsterdam Preoperative Anxiety and Information Scale.

^m^ Evaluation of the Experience of Anaesthesia—Regional Anaesthesia.

^n^ McGill Pain Questionnaire.

**TABLE 2:** Overview of randomised studies contributing to each meta‐analysis outcome.

Study ID	Meta‐analysis anxiety (STAI)	Meta‐analysis anxiety (NRS/VAS)	Meta‐analysis pain (NRS/VAS)	Meta‐analysis satisfaction (NRS)	Meta‐analysis PACU (min/h)
Arifin et al. (2023)	✓				
Boonreunya et al. (2022)			✓		
Bruno et al. (2020)		✓	✓		
Carella et al. (2024)		✓	✓		✓
Faruki et al. (2022)		✓		✓	✓
Fouks et al. (2022)			✓		
Gökçe and Arslan (2023)	✓		✓		
Gray et al. (2021)		✓			
Hecken et al. (2023)		✓	✓	✓	
Ko et al. (2024)	✓		✓	✓	
Lind et al. (2023)	✓	✓	✓		
Liu et al. (2022)			✓		
Luczak et al. (2021)			✓		
Melcer et al. (2021)	✓		✓		
Palte et al. (2024)	✓				
Pelazas‐Hernandez et al. (2023)			✓		
Prabhu et al. (2024)	✓	✓	✓		
Rosielle et al. (2024)			✓		
Sewell et al. (2023)		✓	✓	✓	
Shamali et al. (2024)			✓	✓	
Sooriyaghandan et al. (2023)	✓		✓		
Squara et al. (2024)		✓	✓		
Steinkraus et al. (2024)	✓		✓		
Xu et al. (2024)		✓		✓	

Abbreviations: h, hours; min, minutes; NRA, Numeric Rating Scale; STAI, State‐Trait Anxiety Inventory; VAS, Visual Analogue Scale.

### 3.1 Anxiety

Overall, anxiety was reported in 26 studies, involving 2119 patients. Among these, 11 studies used the Visual Analogue Scale (VAS) or the Numerical Rating Scale (NRS) 13 studies used the State–Trait Anxiety Inventory (STAI), and four studies used validated questionnaires (Almedhesh et al. 2022; Fouks et al. 2022; Rosielle et al. 2024; Soret et al. 2022) (Table 2). The Risk of bias for anxiety, assessed with ROB2, showed three trials with “low risk” of bias, 17 trials with “some concerns”, and one trial with “high risk” of bias. Risk of bias assessed by ROBINS‐I, showed one study with “moderate risk”, two studies with “serious risk”, and one study with “critical risk” of bias.

#### 3.1.1 Anxiety Measured by VAS/NRS

**FIGURE 2:**
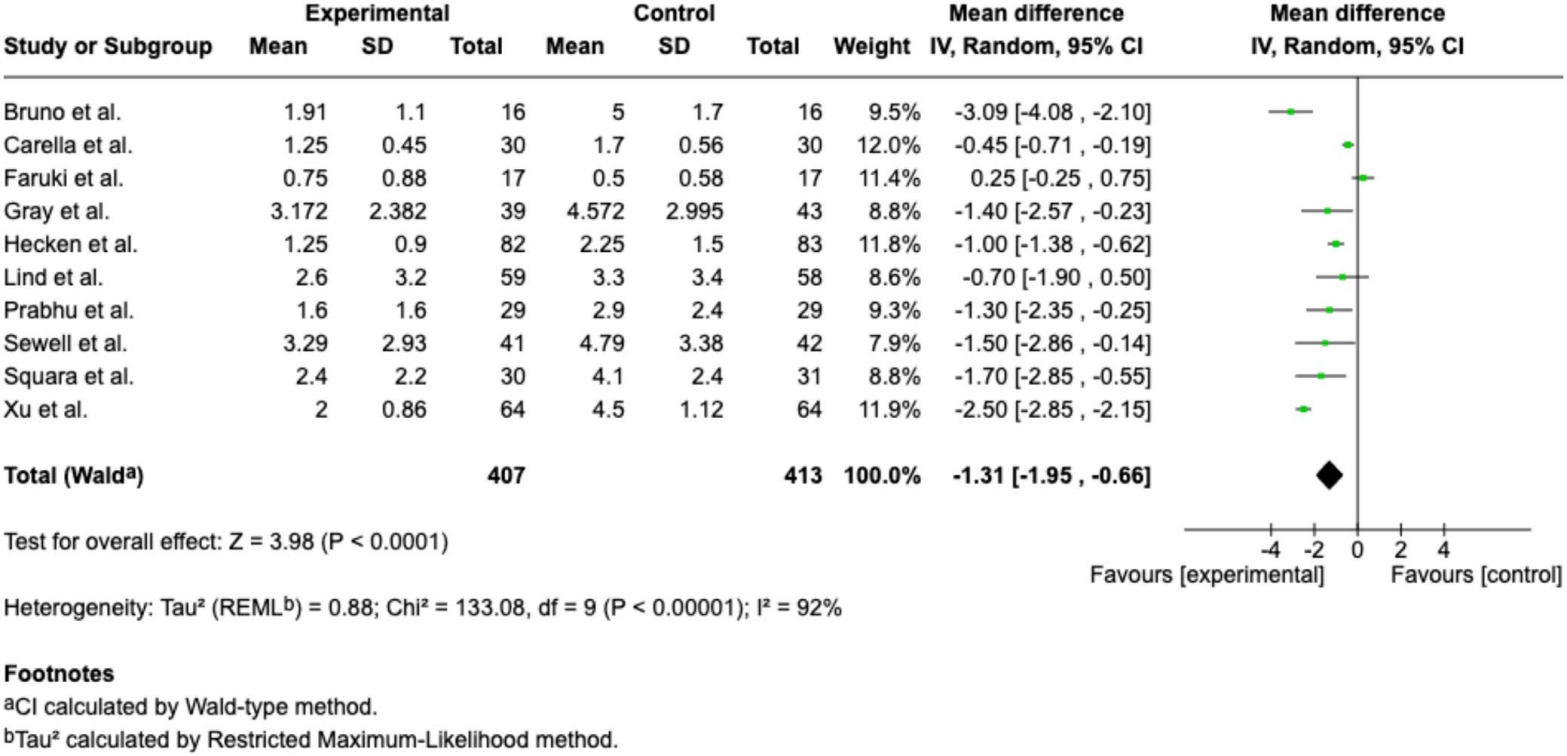
Forest plots of the outcome anxiety, measured by Numeric Rating Scale. CI, confidence interval.

**FIGURE 3:**
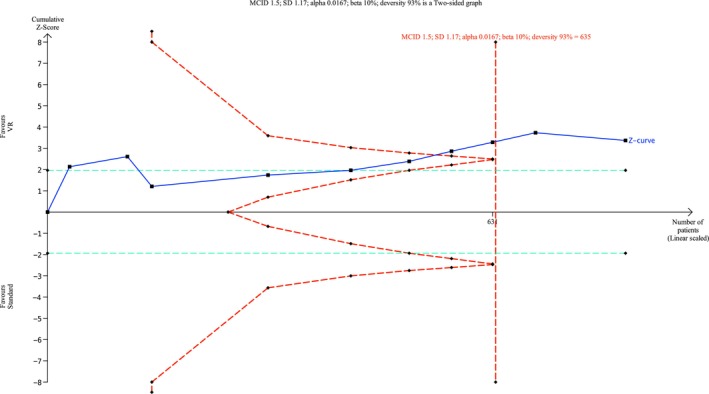
Trial sequential analysis of outcome, anxiety measured by Numeric Rating Scale.

**FIGURE 4:**
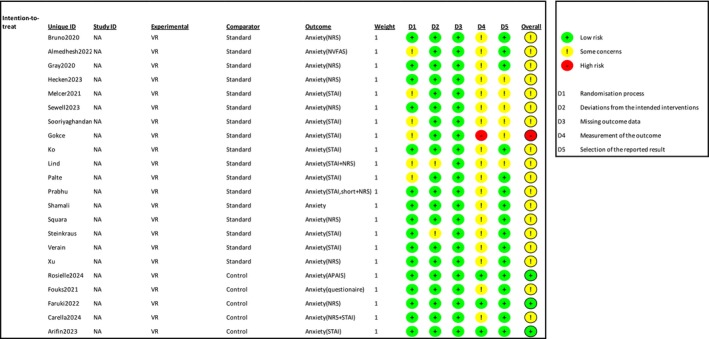
Risk of bias assessment of the included trials for the outcome anxiety. The upper panel presents a study‐level summary of bias judgements across individual domains (green, low risk; yellow, some concerns; red, high risk). The lower panel provides an aggregated overview of risk levels across all domains for the included trials.

**FIGURE 5:**
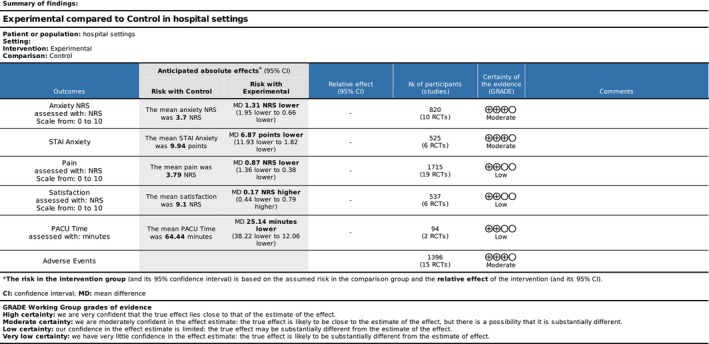
Quality of evidence (GRADE). High certainty: We are very confident that the true effect lies close to that of the estimate of the effect. Moderate certainty: We are moderately confident in the effect estimate: The true effect is likely to be close to the estimate of the effect, but there is a possibility that it is substantially different. Low certainty: Our confidence in the effect estimate is limited: The true effect may be substantially different from the estimate of the effect. Very low certainty: We have very little confidence in the effect estimate: The true effect is likely to be substantially different from the estimate of effect. CI, confidence interval; MD, mean difference.

Ten trials (Bruno et al. 2020; Carella et al. 2024; Faruki et al. 2022; Gray et al. 2021; Hecken et al. 2023; Lind et al. 2023; Prabhu et al. 2024; Sewell et al. 2023; Squara et al. 2024; Xu et al. 2024) and two cohort studies (Alaterre et al. 2020; Lachkar et al. 2022) reported anxiety measured by VAS/NRS, involving 940 patients (Table 2). The meta‐analysis found a significant reduction in anxiety for the VR group compared to usual practice (MD −1.31 NRS, 95% CI: −1.95 to −0.66 NRS, *p* < 0.0001) (Figure 2). TSA adjusted 95% CI: −1.9 to −0.5 NRS, required sample size (DARIS) = 635, *I* ^2^ = 92% (Figure 3). The risk of bias for all trials was “some concerns” (Figure 4), and the quality of evidence (GRADE) was moderate (Figure 5).

#### 3.1.2 Studies Not Included in the Meta‐Analysis

Alaterre et al. (2020) found that patients undergoing upper limb surgery using VR had a statistically significantly lower anxiety score (*p* < 0.001) than the control group, with a “moderate” risk of bias. Lachkar et al. (2022) found a median anxiety level of 9 (out of 10) before bronchoscopy and during the procedure, a median of 4 (out of 10), respectively. The risk of bias ROBINS‐I was “serious”.

#### 3.1.3 Anxiety Measured by STAI

**FIGURE 6:**
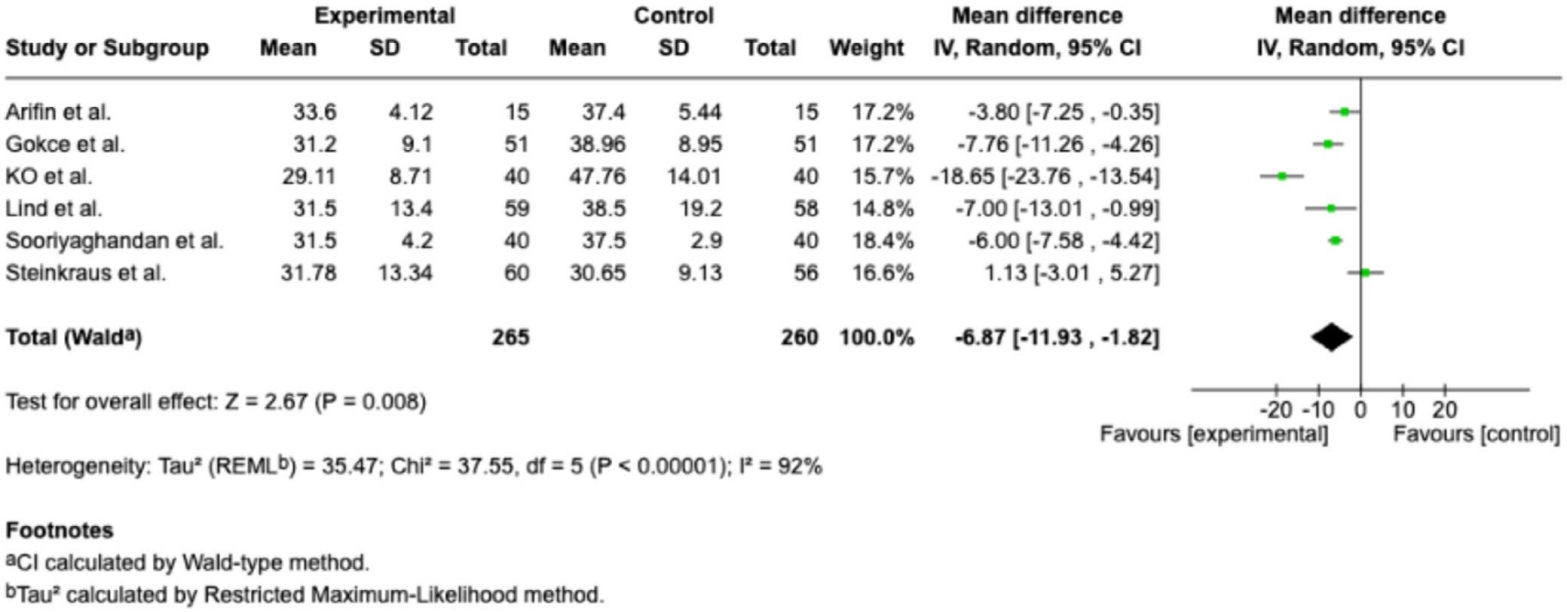
Forest plots of the outcome anxiety measured by State–Trait Anxiety Inventory. CI, confidence interval.

**FIGURE 7:**
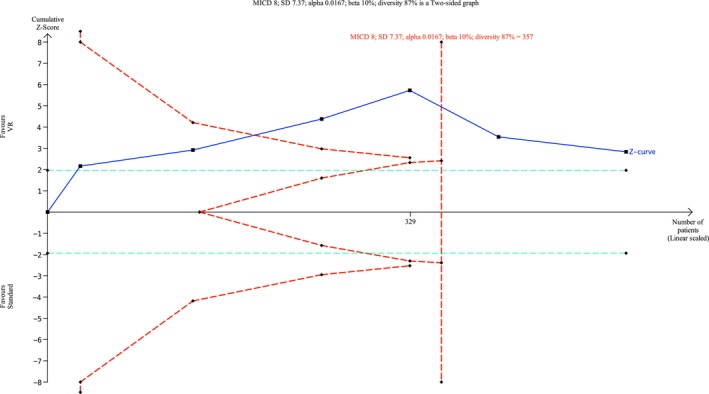
Trial sequential analysis of outcome, anxiety measured by State–Trait Anxiety Inventory.

Eleven trials and two cohort studies (McCullough et al. 2023; Peuchot et al. 2021), involving 974 patients, investigated anxiety measured by STAI (Table 2). The meta‐analysis showed a significant reduction in anxiety for the VR group (MD −6.87, 95% CI: −11.93 to −1.82 points; *p* = 0.008) (Figure 6), TSA‐adjusted 95% CI: −11.54 to −2.05 STAI points, DARIS = 357, *I* ^2^ = 87% (Figure 7). The risk of bias for all trials was “some concerns” (Figure 4), and the quality of evidence (GRADE) was moderate (Figure 5).

#### 3.1.4 Studies Not Included in the Meta‐Analysis

The following six trials (Carella et al. 2024; Melcer et al. 2021; Palte et al. 2024; Prabhu et al. 2024; Shamali et al. 2024; Verain et al. 2024) were not included in the meta‐analysis due to their differing use of the STAI score. Prabhu et al. (2024), who assessed a short version of STAI, found a significant reduction in the VR group for patients undergoing breast biopsy. Risk of bias was “some concerns”. Shamali et al. (2024) found no significant difference in the level of anxiety after colonoscopy. Risk of bias was “some concerns”. No significant differences were reported by Verain et al. (2024), Melcer et al. (2021), and Carella et al. (2024). The trials had all risk of bias deemed as “some concerns”. McCullough et al. (2023) found a significant reduction in anxiety for the VR group during hand surgery. The risk of bias was “serious”. Peuchot et al. (2021) found no significant difference in outcomes for patients undergoing total knee arthroplasty (TKA). Risk of bias was “critical”.

#### 3.1.5 Anxiety Questionnaires

Rosielle et al. (2024) found a significant reduction in anxiety in the VR group assessed with APAIS for women undergoing hysterosalpingography (*p* = 0.018). Risk of bias was “low”. Almedhesh et al. (2022) found a significant (*p* < 0.001) reduction in anxiety assessed by NVFAS in women undergoing caesarean section. The risk of bias showed “some concerns”. Fouks et al. (2022) and Soret et al. (2022) found no significant difference between groups for patients undergoing hysteroscopy and bone marrow aspiration. Risk of bias showed “some concerns” and “serious”, respectively.

### 3.2 Pain

Overall, 29 studies reported outcomes regarding pain, involving 2601 patients. Among these, 26 studies employed VAS (0–100/0–10) or NRS (0–100/0–10). Three studies used the McGill pain score (Palte et al. 2024; Sargut et al. 2024; Soret et al. 2022) (Table 2). Overall risk of bias for pain trials assessed with ROB2 showed 21 with “some concerns”, and three with “high risk” of bias. Studies assessed by ROBINS‐I found four with “serious” and one with “critical” risk of bias.

#### 3.2.1 Pain Measured by NRS

**FIGURE 8:**
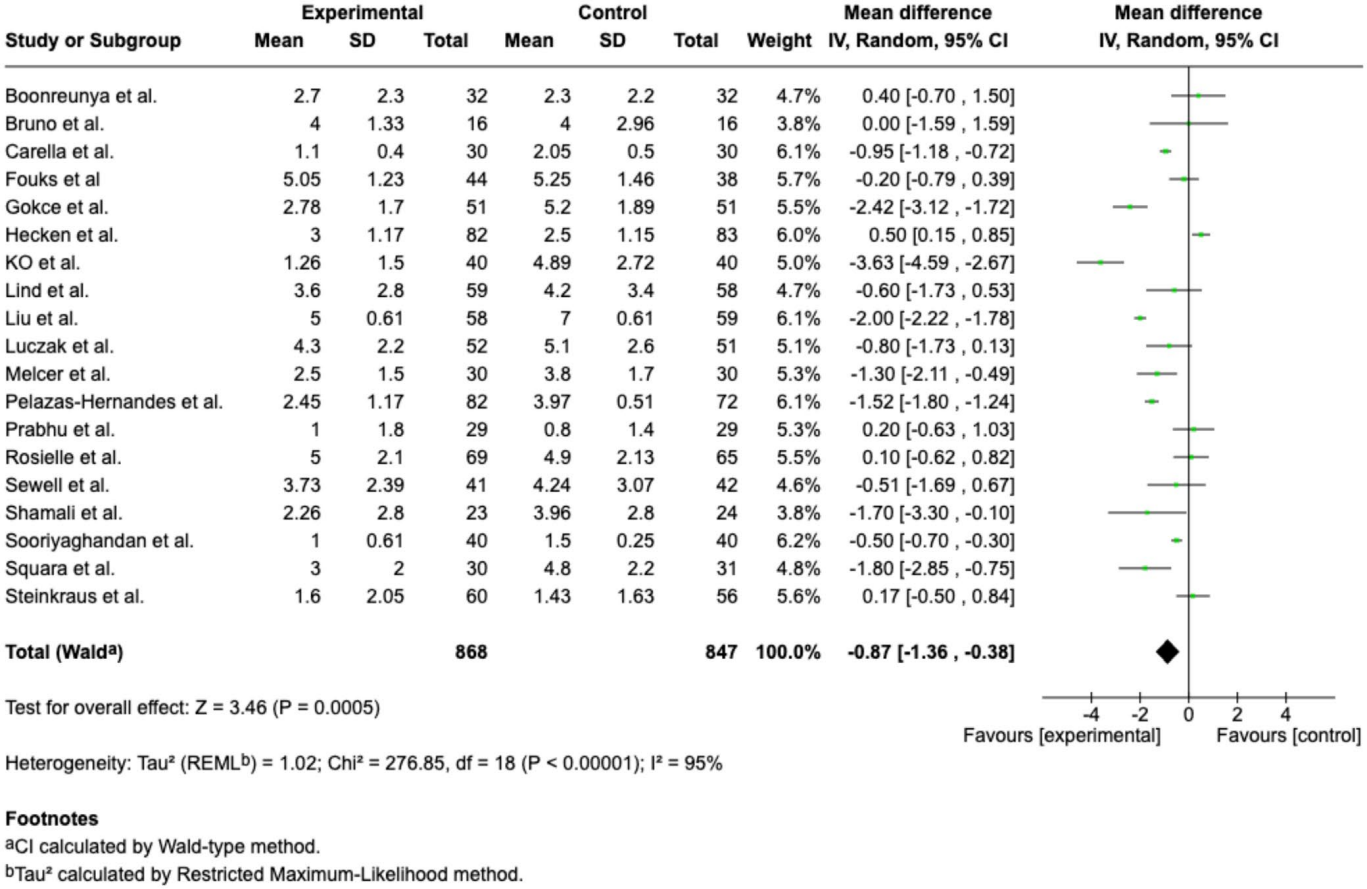
Forest plots of the outcome pain, measured by Numeric Rating Scale. CI, confidence interval.

**FIGURE 9:**
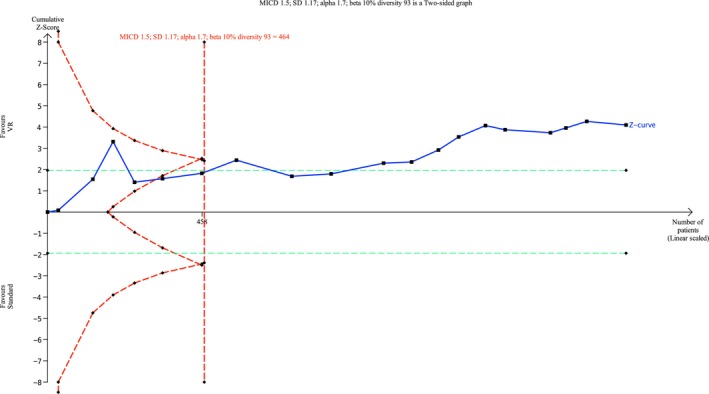
Trial sequential analysis of outcome, pain measured by Numeric Rating Scale.

**FIGURE 10:**
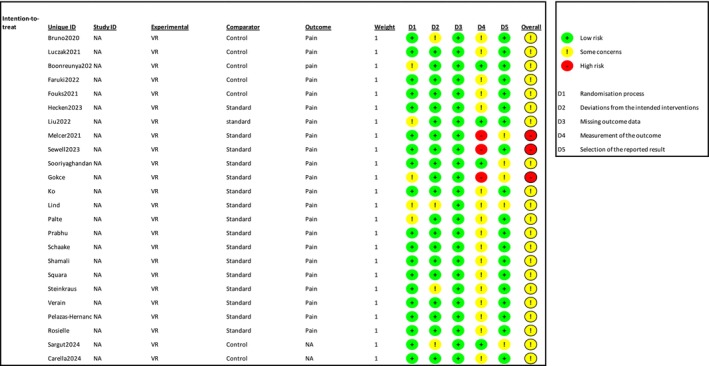
Risk of bias assessment of the included trials for the outcome pain. The upper panel presents a study‐level summary of bias judgements across individual domains (green, low risk; yellow, some concerns; red, high risk). The lower panel provides an aggregated overview of risk levels across all domains for the included trials.

**FIGURE 11:**
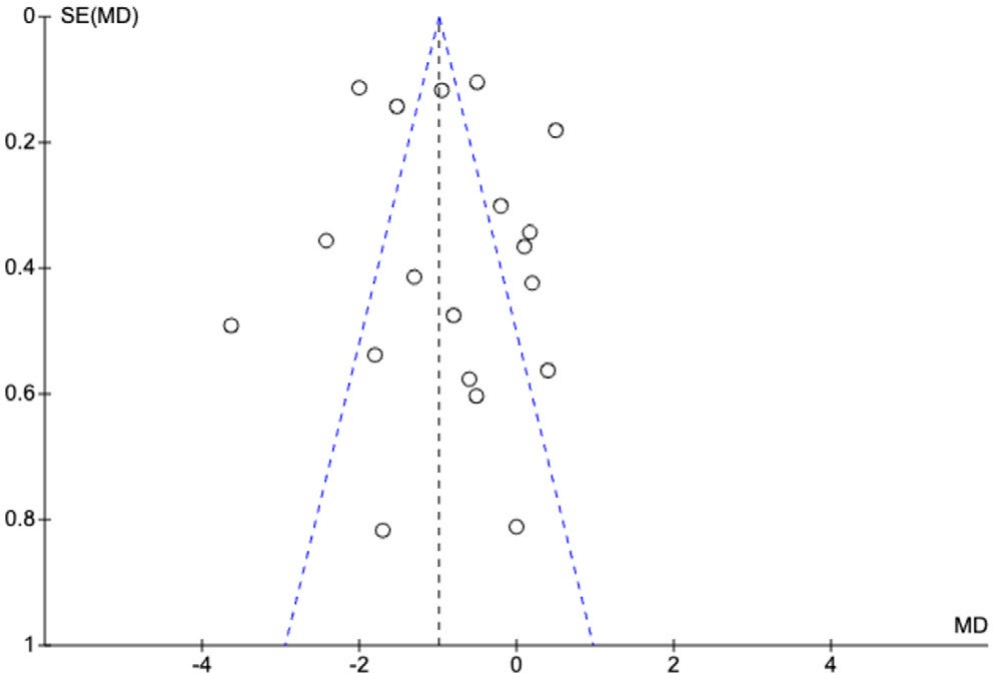
Funnel plot of the outcome pain measured by Numeric Rating Scale.

Twenty trials and four cohort studies (Barry et al. 2022; Perenic et al. 2023; Peuchot et al. 2021; Veisman et al. 2024) reported pain measured by NRS (Liu et al. 2022) (Table 2). The meta‐analysis found a significant reduction in pain for the VR group (MD −0.87 NRS 95% CI: −1.36 to −0.38 NRS, *p* < 0.00005) (Figure 8), TSA‐adjusted 95% CI −1.28 to −0.45, DARIS = 464, *I* ^2^ = 91% (Figure 9). The risk of bias for all trials was “some concerns” (Figure 10), and the quality of evidence (GRADE) was low (Figure 5). To explore the risk of publication bias among trials, a funnel plot was generated (Figure 11). Visual inspection revealed a largely symmetrical distribution of effect estimates around the central mean difference, suggesting a low likelihood of substantial publication bias. A few studies fell outside the funnel's pseudo‐95% confidence limits, which may reflect minor heterogeneity or random variation rather than systematic bias.

#### 3.2.2 Studies Not Included in the Meta‐Analysis

Varain et al. compared VR with sedation for patients undergoing coronary catheterization and found a mean pain NRS of 2.5 for the VR group and NRS of 1.0 for the sedation group. The risk of bias showed “some concerns”. Perenic et al. (2023) compared patients with experience from a former biopsy with patients without former experience and found a statistically significant difference (*p* = 0.013). The risk of bias was “serious”. Peuchot et al. (2021) reported a reduction in median NRS of 2.2 for the VR group compared to standard care (*p* = 0.043) for patients undergoing total knee arthroplasty. The risk of bias was “critical”. Veisman et al. (2024) found that patients in the VR group reported a significantly higher NRS compared to controls (*p* < 0.001) for patients undergoing unsedated colonoscopy. The risk of bias was “serious”. Faruki et al. (2022) found no statistical difference between groups undergoing hand surgery. The risk of bias was “some concerns”.

#### 3.2.3 Pain Measured by McGill

Sargut et al. (2024) found a significant difference in the McGill score in the VR group compared to the control (*p* = 0.028) for patients who had port implantation under local anaesthesia. The risk of bias showed “some concerns”. No significant difference was found in the following studies: Soret et al. (2022); Palte et al. (2024); Schaake et al. (2024), Carella et al. (2024); Faruki et al. (2022), and Barry et al. (2022). Risk of bias was “serious” for two studies (Barry et al. 2022; Soret et al. 2022) and “some concerns” for four studies (Carella et al. 2024; Faruki et al. 2022; Palte et al. 2024; Schaake et al. 2024).

### 3.3 Satisfaction

Overall, 22 studies reported outcomes regarding patient satisfaction postoperatively, involving 2253 patients. Nine studies used VAS (0–100/0–10) or NRS (0–100/0–10) (Table 2), nine used different Likert scales (Arifin et al. 2023; Boonreunya et al. 2022; Gray et al. 2021; McCullough et al. 2023; Prabhu et al. 2024; Rosielle et al. 2024; Sooriyaghandan et al. 2023; Steinkraus et al. 2024; Verain et al. 2024), two used categorical measures (Bruno et al. 2020; Lind et al. 2023), and two used standardised or modified satisfaction tools (Almedhesh et al. 2022; Peuchot et al. 2021). The overall risk of bias for satisfaction, assessed using ROB2, showed that 13 trials had “some concerns” and four trials had a “high risk” of bias. The risk of bias assessed using the ROBINS‐I tool, identified three studies with a “serious risk”, one with a “critical risk”, and one with a “moderate risk” of bias.

#### 3.3.1 Satisfaction Measured by NRS

**FIGURE 12:**
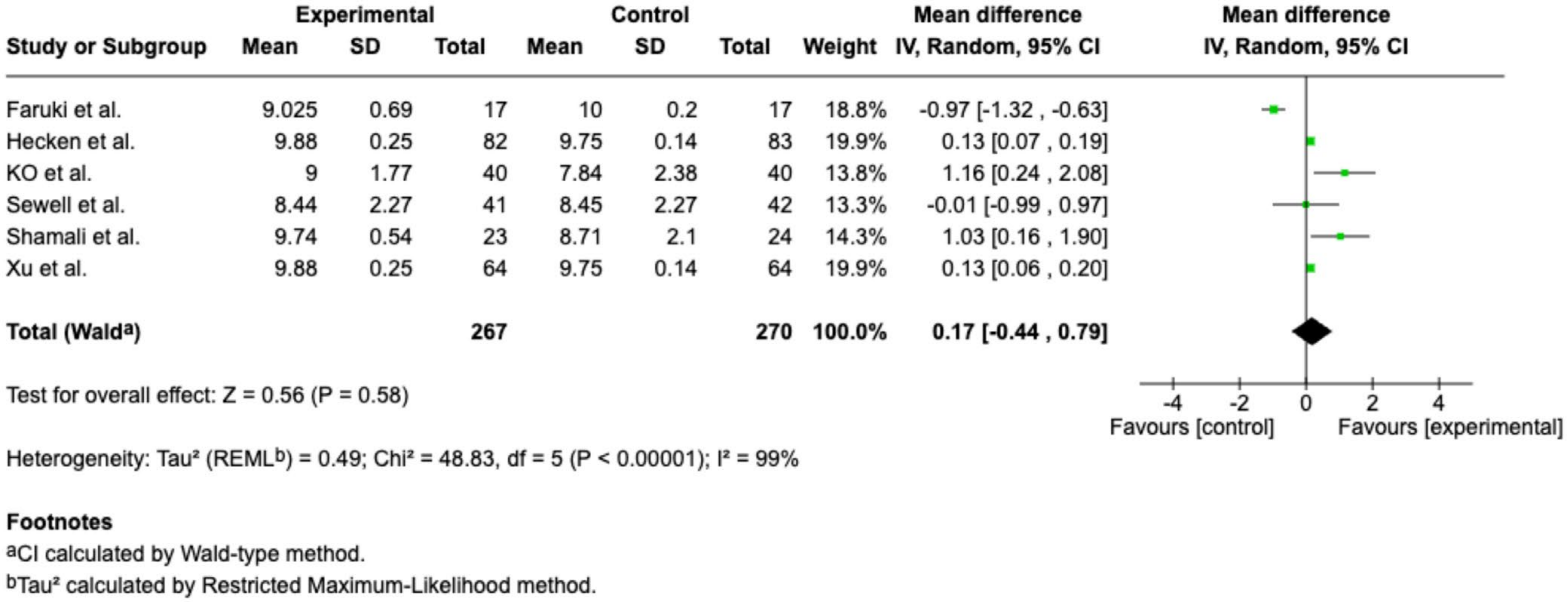
Forest plots of the outcome Satisfaction, measured by Numeric Rating Scale. CI, confidence interval.

**FIGURE 13:**
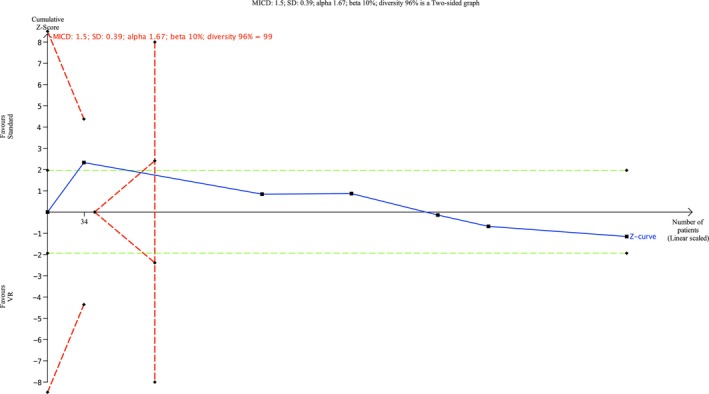
Trial sequential analysis of outcome, satisfaction measured by Numeric Rating Scale.

**FIGURE 14:**
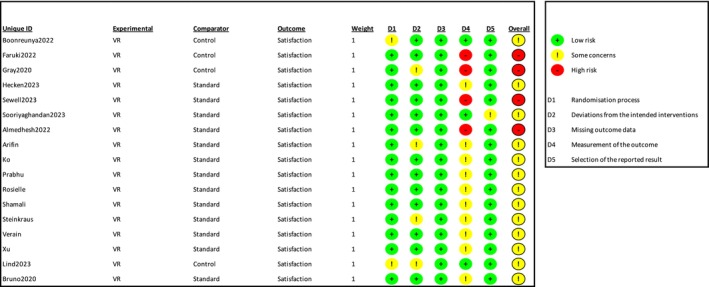
Risk of bias assessment of the included trials for the outcome satisfaction. The upper panel presents a study‐level summary of bias judgements across individual domains (green, low risk; yellow, some concerns; red, high risk). The lower panel provides an aggregated overview of risk levels across all domains for the included trials.

Six trials (Table 2) and three cohort studies (Alaterre et al. 2020; Lachkar et al. 2022; Veisman et al. 2024) reported satisfaction measured using NRS. The meta‐analysis found statistical significance in satisfaction for the VR groups (MD 0.17 NRS, 95% CI: −0.44 to 0.79 NRS, *p* = 0.58 (Figure 12), TSA‐adjusted 95% CI −0,14 to 0.53 NRS, DARIS 99, *I* ^*2*^ = 90%) (Figure 13). The risk of bias for all trials showed “some concerns” (Figure 14), and the quality of evidence (GRADE) was low (Figure 5).

#### 3.3.2 Studies Not Included in the Meta‐Analysis

Veisman et al. (2024) found no difference between groups in a cohort of colonoscopy patients. Risk of bias was “serious”. Lachkar et al. (2022) found high satisfaction (10 out of 10) with VR among patients and staff during bronchoscopy. Risk of bias was “serious”. Alaterre et al. (2020) found significantly higher satisfaction (*p* < 0.001) in the VR group for upper limb surgery under peripheral nerve block. Risk of bias was “moderate”.

#### 3.3.3 Satisfaction Measured on a Likert Scale

Verain et al. (2024) found that 96.7% of patients were satisfied in the VR group compared to 100% in the sedation group. Additionally, if the procedure had to be repeated, 88.3% of patients in the VR group reported that they would like to try VR again. Among the 41 patients in the VR group who had previously undergone cardiac catheterization without VR therapy, 87.7% preferred the procedure with VR therapy. Risk of bias was “some concerns”. Sooriyaghandan et al. (2023) found a statistically significant difference in favour of the VR groups for patients undergoing flexible bronchopy (*p* < 0.001). Risk of bias showed “some concerns”. Prabhu et al. (2024) found 85.7% of patients reported “very satisfied,” and 10.7% reported “satisfied” with the VR headset and the intervention. Only 3.6% (1 out of 28) reported a “neutral” feeling, and none of the patients selected being “dissatisfied” or “very dissatisfied.” Additionally, 89.3% were “very likely” to recommend the VR intervention to others undergoing biopsy procedures. Risk of bias was “some concerns”. Arifin et al. (2023) found a significantly higher satisfaction (*p* = 0.033) in the VR group for patients undergoing regional anaesthetic surgery. Risk of bias was “some concerns”. McCullough et al. (2023) reported a postprocedural satisfaction score of 4.3 on a Likert scale (one to five [most satisfied]) for patients undergoing wide‐awake local‐only hand surgery. Risk of bias was “serious”. Gray et al. (2021); Boonreunya et al. (2022); Rosielle et al. (2024); Steinkraus et al. (2024) found no significant difference between groups. Risk of bias was “high” for one study (Gray et al. 2021) and “some concerns” for three studies (Boonreunya et al. 2022; Rosielle et al. 2024; Steinkraus et al. 2024).

#### 3.3.4 Satisfaction by Categorical Measures

Bruno et al. (2020) found that 93.8% of patients preferred using VR in future medical procedures. Risk of bias was “some concerns”. Lind et al. (2023) evaluated patient satisfaction by inquiring whether participants during transcatheter aortic valve implantation under local anaesthesia would recommend the use of VR headsets to others. A total of 88.9% of respondents indicated that they would endorse the use of VR. Risk of bias was “some concerns”.

#### 3.3.5 Satisfaction Measured by a Standardised Or Modified Tool

Almedhesh et al. (2022) found that satisfaction with the overall delivery process was significantly higher in the VR group during caesarean sections. More than half of the patients in the VR group were completely satisfied, compared to one‐tenth of patients in the control group, measured by the Birth Satisfaction Scale‐Revised. Risk of bias was “high”. Peuchot et al. (2021) found no significant difference between groups undergoing total knee arthroplasty, measured by the EVAN LR satisfaction score. Risk of bias was “critical”.

### 3.4 PACU Time

Overall, five studies reported PACU/recovery time (Alaterre et al. 2020; Barry et al. 2022; Carella et al. 2024; Faruki et al. 2022; Veisman et al. 2024), involving 314 patients (Table 2). The risk of bias measured by ROB2 found one trial with “some concerns” and one trial with “high risk” of bias. Risk of bias assessed by ROBINS‐I showed two studies with “serious”, one with “moderate”.

#### 3.4.1 Observation Time

**FIGURE 15:**
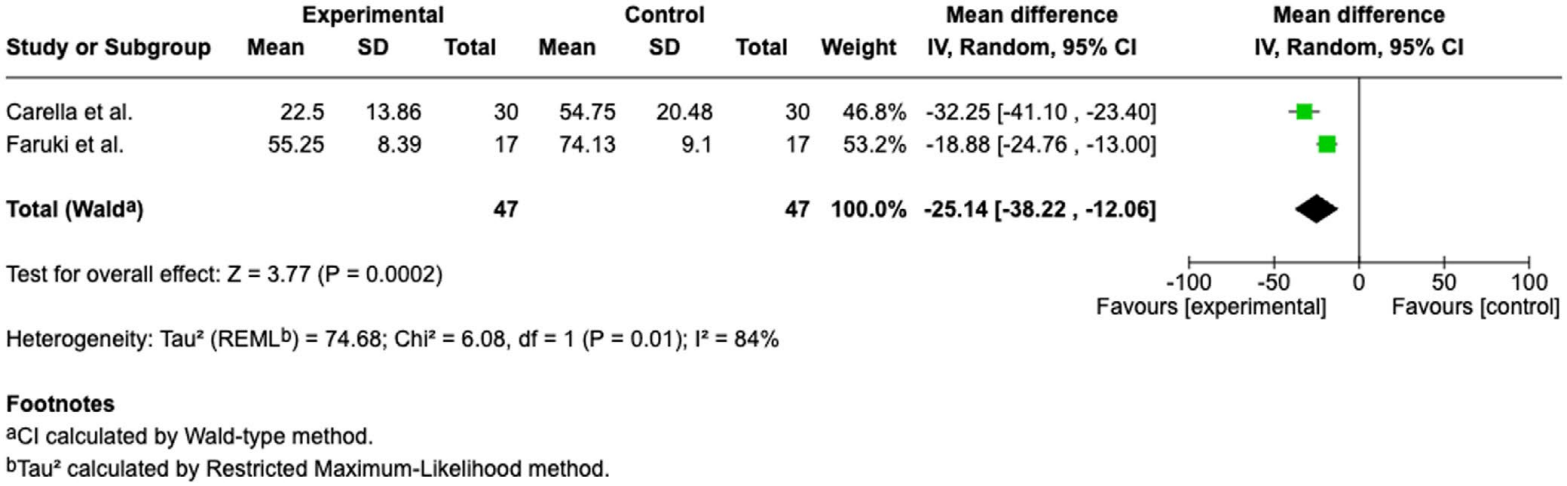
Forest plots of the outcome PACU time, measured by minutes. CI, confidence interval.

**FIGURE 16:**
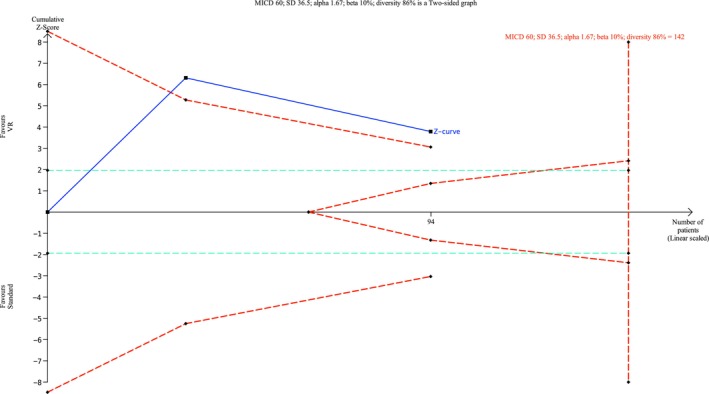
Trial sequential analysis of outcome, PACU time measured in minutes. MCID = 60 min.

**FIGURE 17:**
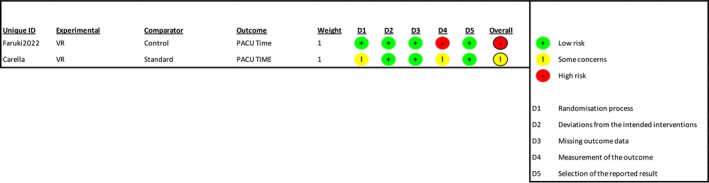
Risk of bias assessment of the included trials for the outcome PACU time. The upper panel presents a study‐level summary of bias judgements across individual domains (green, low risk; yellow, some concerns; red, high risk). The lower panel provides an aggregated overview of risk levels across all domains for the included trials.

The meta‐analysis found a significant reduction in PACU time for the VR group (MD = −25.14 min., 95% CI −38.22 to −12.06 min, *p* = 0.0002) (Figure 15). TSA‐adjusted 95% CI: −38.22 to 12.06 min, DARIS = 142, *I* ^2^ = 84% (Figure 16). The risk of bias for all trials was “high risk” (Figure 17), and the quality of evidence (GRADE) was low (Figure 5).

#### 3.4.2 Studies Not Included in the Meta‐Analysis

Three cohort studies (Alaterre et al. 2020; Barry et al. 2022; Veisman et al. 2024) investigated the impact of VR on recovery time. The results from Veisman et al. (2024) found a significant difference in favour of the VR group (*p* < 0.001) for patients undergoing colonoscopy. Risk of bias was “serious”. Alaterre et al. (2020) and Barry et al. (2022) found no significant difference for patients undergoing upper limb surgery or Primary Total Hip and Knee Arthroplasty. Risk of bias was “moderate” and “serious”.

### 3.5 Adverse Events

**FIGURE 18:**
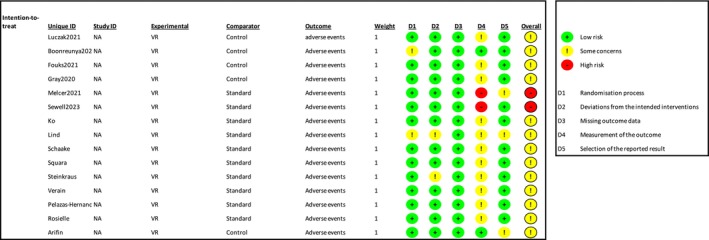
Risk of bias assessment of the included trials for the outcome adverse Events. The upper panel presents a study‐level summary of bias judgements across individual domains (green, low risk; yellow, some concerns; red, high risk). The lower panel provides an aggregated overview of risk levels across all domains for the included trials.

Thirteen studies reported the following adverse events: dizziness, cybersickness, nausea, or other adverse events (Alaterre et al. 2020; Arifin et al. 2023; Barry et al. 2022; Fouks et al. 2022; Lind et al. 2023; Luczak et al. 2021; Pelazas‐Hernandez et al. 2023; Peuchot et al. 2021; Rosielle et al. 2024; Sewell et al. 2023; Squara et al. 2024; Steinkraus et al. 2024; Veisman et al. 2024) (Table 2). The overall risk of bias for adverse events, assessed using ROB2, showed 13 with “some concerns” and two with “high risk” of bias. Risk of bias assessed by ROBINS‐I showed three studies with “serious”, one with “moderate”, and one with “critical” risk of bias (Figure 18). The quality of evidence (GRADE) was moderate (Figure 5). Square et al. (20) found, in the VR group, four out of 30 (13%) patients ended their VR session due to cybersickness. Risk of bias was “some concerns”. Pelazas‐Hernandez et al. (2023) reported four patients experiencing dizziness during hysteroscopy. Three patients from the control group (3.6%) and one from the intervention group (1.4%). Risk of bias was “low”. Luczak et al. (2021) found increased nausea in the VR group NRS 1.8 vs. NRS 1.1 in the control group for patients undergoing cystoscopy. Risk of bias was “some concerns”. Peuchot et al. (2021) found a significant decrease in blood pressure in the VR group for patients undergoing total knee arthroplasty. Risk of bias was “critical”. Alaterre et al. (2020) found no adverse events related to VR for upper limb surgery with “moderate” risk of bias. All other studies found no significant difference in adverse events between groups (Arifin et al. 2023; Barry et al. 2022; Fouks et al. 2022; Lind et al. 2023; Rosielle et al. 2024; Sewell et al. 2023; Steinkraus et al. 2024; Veisman et al. 2024). Risk of bias was assessed as “moderate”.

### 3.6 Serious Adverse Events

Seven studies reported on serious adverse events (Boonreunya et al. 2022; Gray et al. 2021; Ko et al. 2024; Melcer et al. 2021; Perenic et al. 2023; Schaake et al. 2024; Verain et al. 2024) and did not find any (Table 2). Risk of bias for five studies was “some concerns” (Boonreunya et al. 2022; Gray et al. 2021; Ko et al. 2024; Schaake et al. 2024; Verain et al. 2024). One study was “high risk” (Melcer et al. 2021) and one “serious” risk of bias (Perenic et al. 2023).

## 4 Discussion

This systematic review with meta‐analysis and trial sequential analysis demonstrated with moderate certainty that VR significantly reduced anxiety, as measured by the NRS and STAI, and with low certainty that pain, as measured by the VAS/NRS and McGill's pain scale, was also reduced. Patient satisfaction improved, and the recovery time was shorter. Adverse events were infrequent and mild, with no serious adverse events reported. The risk of bias was primarily characterized as having “some concerns,” and the evidence certainty ranged from moderate to low. Patient readiness and surgical delay were not detected in the included studies. To enhance the robustness of the findings, the meta‐analysis was supported by Trial Sequential Analysis. It should be noted that VR was only used for a short period in the included studies (6–120 min), and the most beneficial duration for VR use was not within the scope of this review. Most included studies had “some concerns” to “high” risk of bias and moderate to low certainty of the evidence. Therefore, our results should be interpreted with caution. Furthermore, the Minimal Clinically Important Difference (MCID) of 15 mm was only obtained for outcomes regarding anxiety and not pain. Among the studies that were not included in the meta‐analysis, we found equal evidence between those showing a significant reduction in anxiety and those showing no reduction. The cohort studies dealing with pain outcomes aligned with the overall direction of the randomized trials. The differences in the effectiveness of VR for pain and anxiety reduction, might be explained by the levels of anxiety and pain felt by the patients in advance, the heterogeneity of the research methods such as the use of sedatives in the control group, the type and duration of the procedure, or the patient's capacity to engage with the virtual content. Another issue that needs to be addressed is the strong correlation between anxiety and pain. Patients with higher levels of anxiety preoperatively tend to report more severe pain postoperatively, suggesting a possible interaction between affective and sensory responses in surgical settings (Husni et al. 2024; Shebl et al. 2025). Also, the differences in VR hardware and content delivery methods varied widely, from simple 2D displays to fully immersive 3D environments, with different resolution quality and patient comfort with the VR technology could potentially influence outcomes, as suggested by Hoffman et al. (2006).

Our findings showed a significant difference in patients' level of anxiety in favor of VR and obtained MCID for elective surgical procedures. It is well proven in numerous studies, especially when it comes to fear of blood, needles, injections, and dental treatments, that VR has promising results in reducing anxiety in both adults and children (Triviño‐Martínez et al. 2025). Furthermore, in alignment with our findings, Li et al. (2011) and Gao et al. (2020) find a substantial benefit for preoperative anxiety using VR for elective surgery in their literature reviews, which only include RCTs (Rosa et al. 2023). Their findings reveal considerable heterogeneity, and both conclude that further research is necessary to determine for whom, and which surgical procedures VR is most beneficial. For example, Chatterjee et al. find no change in procedural anxiety, pain, and satisfaction during transcatheter aortic valve replacement when using VR, most likely due to the absence of headphones, which did not provide the immersive experience.

This also addresses the challenges of obtaining the most beneficial experience; the VR hardware needs to be comfortable for patients and provide the best possible immersive experience, to serve as a suitable distraction. This should be supported by the use of sound‐cancelling headphones and adequate sound support that enhances the overall experience (Bosman et al. 2024). A systematic review by Kakar et al. (2021), found that interventions solely using music for patients after cardiac surgery significantly decrease anxiety. This finding could suggest that the auditory experience might be of even greater importance than the visual experience in terms of reducing patient anxiety.

In line with our findings Malik et al. (2024), underpin in their review of the current evidence regarding VR and postoperative pain management that most studies show that VR can safely and effectively reduce postoperative pain after various surgeries, offering a drug‐free supplement to standard pain management. However, research is limited by small sample sizes and a lack of studies on different VR features, such as immersion levels and user controls. Although VR showed a modest reduction in postoperative pain, the MCID was not achieved. VR may therefore be interpreted to serve as an adjunctive, but not an alternative, to opioid‐based analgesia.

Furthermore, we found that adverse events associated with VR were typically mild and mostly reported as nausea in the term of cybersickness and no serious adverse events were reported in any of the included studies indicating a safe alternative. Another aspect of a possible benefit of VR is patient satisfaction. We found no significant difference in favour of VR. When incorporating studies that fall outside the scope of the meta‐analyses, a similar pattern of inconsistency emerges. Existing literature reports similar findings that satisfaction is a heterogeneous outcome and concludes that further studies need to be performed (Wang et al. 2022). Finally, the ability to decrease the recovery time with VR was found mostly in PACU. Our finding indicates that the effect may be clinically relevant, but the current evidence remains inconclusive and requires further confirmation.

## 5 Strengths and Limitations

This systematic review possesses several notable methodological strengths. It features a comprehensive and rigorously designed search strategy, which reduces the likelihood of missing relevant studies. The review protocol was pre‐registered with PROSPERO, ensuring transparency and methodological rigour. Another methodological strength is the use of TSA as an exploratory sensitivity tool to evaluate the robustness of our findings. For outcomes such as pain and satisfaction, the futility boundaries were crossed, suggesting that additional trials are unlikely to substantially change the overall direction of the evidence. This adds confidence to the interpretation of these results. However, the application of TSA in this review also carries important limitations. TSA was conducted retrospectively in a non‐sequential dataset, which reduces its inferential power. In addition, the assumptions underlying TSA (e.g., anticipated intervention effect, control event rate, and variance estimates) may be unstable in the context of heterogeneous and relatively few included trials. Therefore, while TSA provides a safeguard against random errors and adds methodological rigour, its findings should be regarded as complementary to conventional meta‐analytic estimates and the risk of bias assessment using the Cochrane Risk of Bias tool (Sterne et al. 2016). This interpretative use of TSA has been discussed previously in the evidence synthesis literature (Jakobsen et al. 2014). The certainty of the evidence was systematically appraised with the GRADE framework, providing a clear and structured interpretation of the results. Additionally, the inclusion of cohort studies further supported the review's findings.

However, several limitations must be acknowledged. A primary concern is the high risk of bias present in many of the included trials, mainly due to inadequate reporting of key methodological aspects such as blinding, randomization, and outcome reporting. This widespread risk of bias undermines the reliability of the findings and complicates the assessment of treatment efficacy. As a result, while some interventions demonstrated statistical significance, the clinical relevance and generalizability of these effects remain unclear. Methodological issues, small sample sizes, lack of blinding, and considerable heterogeneity across studies frequently necessitated GRADE downgrading, further challenging the validity of the results. Moreover, comparator groups often received sedation, meaning the true comparison was between VR and sedation, which may have influenced the outcomes. More research is needed, especially for elderly patients and those with special needs, as well as for those with sensory impairments or psychiatric conditions, who may not be suitable for VR. Larger trials are necessary to confirm VR's effectiveness, impact on; for example, opioid use, and cost‐effectiveness. Clear technical standards and guidelines are also needed for VR's integration into postoperative care. These uncertainties have important implications for clinical practice. While several outcomes appeared beneficial, clinicians should interpret these findings with caution. Robust evidence is still lacking regarding which patient populations, surgical procedures, and VR technologies are most likely to benefit.

## 6 Conclusion

This systematic review's findings demonstrate that in‐hospital VR used for elective surgery is feasible and has potential for effectively reducing anxiety, pain, and time in PACU, and can safely be used as a non‐pharmacological intervention with few adverse events. The most robust and clinically supported effect was found for anxiety, with evidence demonstrating consistency and statistical significance at a moderate level of certainty. For pain and satisfaction outcomes, TSA indicated that additional small‐scale trials are unlikely to substantially alter the overall conclusions. However, studies with small sample sizes, a high risk of bias, and questionable methodology limit the certainty of the current evidence. Therefore, additional rigorous and adequately powered clinical trials are still needed to establish the clinical effectiveness and cost‐effectiveness of VR interventions. Furthermore, identifying which surgical procedures and patient groups benefit most from VR will be essential to ensure its effective and appropriate integration into perioperative care.

## Author Contributions

This study was designed by K.L.L., P.J., C.S., A.G. The data extraction was performed by K.L.L., K.H., N.W., and C.S. The data were analysed by K.L.L., K.H. and A.G., and the results were critically examined by all authors. K.L.L. had a primary role in preparing the manuscript and K.H. the secondary role, which was edited by K.L.L., A.G. C.S., and P.J., K.H, A.A. All authors have approved the final version of the manuscript and agree to be accountable for all aspects of the work.

## Conflicts of Interest

The authors declare no conflicts of interest.

## Supporting information


**Appendix S1:** Search strategy.

## Data Availability

The data that support the finding of this systematic review are available from the corresponding author upon reasonable request.
