# Supplementary material for: Virtual Reality Is Safe and Can Reduce In‐Hospital Anxiety and Pain: A Systematic Review With Meta‐Analyses and Trial Sequence Analyses
Source: Eur J Pain. 2025 Nov 5;29(10):e70165. doi: 10.1002/ejp.70165 (PMC12589719; doi:10.1002/ejp.70165)
Supplement: Supplementary file 1 — Appendix S1: Search strategy. [file EJP-29-0-s001.docx]

# Appendix 1. Search strategy

## CINAHL

Dates: 24-05-2023 and 19-11-2024

Total hits: 3637

|  | **Expanders** - Apply equivalent subjects  **Limiters** - Publication Date: 20170101-20231231; Language: Danish, English, Finnish, Norwegian, Swedish  **Search modes** – Proximity  ( virtual reality therap* OR virtual reality interface OR VR interface OR VR system OR virtual reality system ) OR AB ( virtual reality therap* OR virtual reality interface OR VR interface OR VR system OR virtual reality system ) |
| --- | --- |
|  | ( virtual reality therap* OR virtual reality interface OR VR interface OR VR system OR virtual reality system ) OR AB ( virtual reality therap* OR virtual reality interface OR VR interface OR VR system OR virtual reality system ) |
|  |  |
|  |  |
|  |  |
|  | ( virtual reality therap* OR virtual reality interface OR VR interface OR VR system OR virtual reality system ) OR AB ( virtual reality therap* OR virtual reality interface OR VR interface OR VR system OR virtual reality system ) |
|  | (MH "Virtual Reality") |

## Medline

Dates: 24-05-2023 and 19-11-2024

Total hits: 20764

| virtual reality/ |  |
| --- | --- |
| 2 | virtual reality.ab,kf,ti. |
| 3 | 1 or 2 |
| 4 | (Virtual Reality Immersion Therap* or Virtual Reality Therap*).ab,kf,ti. |
| 5 | 3 or 4 |
| 6 | vr.ab,kf,ti. |
| 7 | 5 or 6 |
| 8 | artificial reality.ab,kf,ti. |
| 9 | 7 or 8 |
| 10 | limit 9 to yr="2017 -Current" |

## EMBASE

Embase <1996 to 2023

Dates: 24-05-2023 and 19-11-2024

Total hits: 24645

virtual reality/
virtual reality.ab,kf,ti.
1 or 2
(Virtual Reality Immersion Therap* or Virtual Reality Therap* or virtual reality interface or VR interface or VR system or virtual reality system).ab,kf,ti.
3 or 4
limit 5 to yr="2017 -Current"

## Google Scholar

Google Scholar supplemented the search. The first 500 hits were taken into consideration.

Dates: 24-05-2023 and 19.11.24

Total hits: 492

Virtual Reality Immersion Therapy or virtual reality interface or VR interface or VR system.
